# Optical coherence tomography features of the repair tissue following RPE tear and their correlation with visual outcomes

**DOI:** 10.1038/s41598-021-85270-x

**Published:** 2021-03-16

**Authors:** Francesco Romano, Salvatore Parrulli, Maurizio Battaglia Parodi, Marco Lupidi, Matteo Cereda, Giovanni Staurenghi, Alessandro Invernizzi

**Affiliations:** 1 Eye Clinic, Department of Biomedical Sciences, Luigi Sacco Hospital, University of Milan, Milan, Italy; 2 Department of Ophthalmology, Scientific Institute San Raffaele, Vita-Salute University, Milan, Italy; 3 Department of Surgical and Biomedical Sciences, Section of Ophthalmology, University of Perugia, S. Maria Della Misericordia Hospital, Perugia, Italy; 4 Faculty of Health and Medicine, Save Sight Institute, University of Sydney, Sydney, NSW Australia; 5 Eye Clinic, Department of Biomedical and Clinical Sciences, Luigi Sacco Hospital, University of Milan, Via G.B. Grassi, 74, 20157 Milan, Italy

**Keywords:** Eye diseases, Macular degeneration, Retinal diseases, Vision disorders

## Abstract

To assess the optical coherence tomography (OCT) features of the repair tissue after retinal pigment epithelial (RPE) tear in neovascular age-related macular degeneration. Retrospective, observational study. Medical and imaging records of patients that developed tears after starting anti-VEGF treatment and with at least 12 months of follow-up were reviewed. OCT reflectivity of the RPE-subretinal hyperreflective tissue (SHT) complex was measured at 6, 12 and 18 months (when available). Reflectivity of the adjacent unaffected RPE-Bruch’s membrane was taken as internal reference. Other variables: grade and rip occurrence (early/late); number of intravitreal injections; type of macular neovascularization; sub-macular hemorrhage (SMH) at onset. Forty-nine eyes (age: 76.1 ± 7.0 years; VA: 0.54 ± 0.27 LogMAR) were included. Thirty-eight eyes had OCT signs of healing during the follow-up, with 21 showing SMH at baseline. Final VA positively correlated with the number of injections and negatively correlated with the RPE-SHT reflectivity and the presence of SMH (*p* < 0.001). Reflectivity of the RPE-SHT complex was positively associated with time and SMH at baseline (*p* < 0.05). In our study, most eyes showed signs of tissue repair after RPE tear. The reflectivity of repair tissue, the SMH presence and the number of anti-VEGF injections appeared to be major predictors of visual outcomes.

## Introduction

Tears of the retinal pigment epithelium (RPE) are a well-known complication of neovascular age related macular degeneration (nAMD) that result from a breaking of the RPE layer under the effect of significant tangential forces^1–4^. Retinal pigment epithelium tears are more frequent in nAMD eyes characterized by vascularized pigment epithelium detachment (PED) and can occur spontaneously or following intravitreal anti-vascular endothelial growth factor (VEGF) injections^5–7^.

RPE tears are associated with a drop in visual acuity that can be limited only in part by the available treatments^8–11^. However, some eyes with RPE tears seem to undergo some sort of self-healing process over the months following the tear. Resurfacing of the denuded area by means of RPE proliferation and appearance of a sub-retinal hyperreflective tissue (SHT) plaque have been proposed as possible mechanisms for RPE tears repair in these eyes^12–17^.

Optical coherence tomography (OCT) is an imaging technique that allows to evaluate the extent of the tear and to characterize the structural features of the RPE, the healing tissue and the overlying retina. Numerous OCT studies have identified anatomical risk factors for RPE tear occurrence, but only few have focused on the healing process and its possible correlations with patients’ outcomes^18–22^.

The aim of this study was to analyze the OCT features characterizing the healing process in eyes that experienced a RPE tear during anti-VEGF treatment for nAMD and to investigate their possible correlations with functional outcomes.

## Materials and methods

The study was a retrospective, multicenter, observational study. Ethics approval was obtained from the institutional review boards of the involved centers. All patients provided informed consent in a written form and the procedures adhered to the tenets of the Declaration of Helsinki.

Medical records and imaging studies of patients affected by nAMD and followed at three referring centers (Eye Clinic, Luigi Sacco Hospital, University of Milan, Milan, Italy; Department of Ophthalmology of Scientific Institute San Raffaele, Milan, Italy; and Eye Clinic, University of Perugia, Perugia, Italy) from September 2011 to January 2019 were systematically reviewed.

To be included in the analysis all eyes had to fulfill the following criteria: (1) angiography-confirmed diagnosis of nAMD undergoing treatment with anti-VEGF intravitreal injections, (2) best-corrected visual acuity (BCVA) higher than 1.0 LogMAR (approximately 20/200 Snellen equivalents) at the time of nAMD diagnosis, (3) the development of an RPE tear after the initiation of the anti-VEGF treatment, (4) a minimum of 12 months follow-up after the tear, (5) consecutive OCT raster scans comprising the whole area of the RPE tear and performed with the Spectralis OCT (Heidelberg Engineering, Heidelberg, Germany) using the “follow-up” function, which automatically re-acquires the scans exactly in the same location at each visit, (6) blue-light fundus autofluorescence (BAF; exciting at λ = 488 nm, emitting at 500–700 nm; 30° × 30° field of view) performed at each follow-up.

Exclusion criteria included: (1) any ocular or systemic disorder known to cause retinal alterations and consequently able to affect our analysis, (2) refractive errors greater than ± 3D (spherical equivalent) in order to limit the angle-dependent optical variability of OCT signal^23,24^, (3) any ocular surgery other than intravitreal injections during the follow-up period, (4) poor quality images.

The diagnosis of nAMD was based on the evidence of macular neovascularization (MNV) with signs of active leakage on fluorescein and indocyanine green angiography. All patients received a loading dose of 3 intravitreal anti-VEGF injections and then were re-assessed on a monthly basis and re-injected as needed (PRN). The MNV was considered to be active based on: appearance/persistence of intra-retinal or sub-retinal fluid, growth and/or undefined margins of the PED, presence of sub-retinal hyperreflective material and, when performed, leakage on retinal angiography.

**Figure 1:**
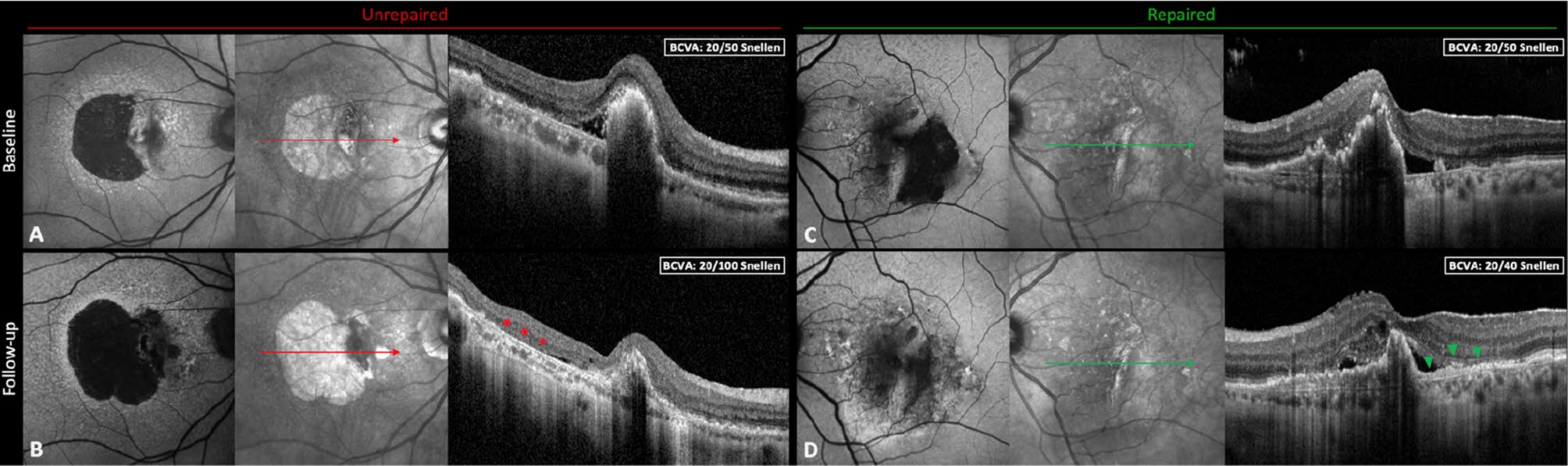
Multimodal imaging of unrepaired vs. repaired tears of the retinal pigment epithelium (RPE). (**A**) and (**B**) composites depict the imaging features of a case of large RPE tear without signs of tissue repair. Over the follow-up the hypo-autofluorescence due to the absence of the RPE gradually enlarges as documented by blue-light autofluorescence (BAF), while the retina rests on an area of exposed Bruch’s membrane on optical coherence tomography (OCT; red asterisks). On the other hand, (**C**) and (**D**) composites show a case characterized by RPE healing. At follow-up, shrinkage of the RPE defect can be noticed using BAF, and the growth of the RPE layer with an underlying proliferative tissue is visible on OCT (green arrowheads).

RPE tears were diagnosed based on typical appearance on multimodal imaging including color fundus photographs, retinal angiography, blue-light autofluorescence (BAF) and OCT. Repair of the tear was defined as the centripetal growth of a new RPE-like layer above the Bruch’s membrane from the border of the tear or as the development of a thick proliferative tissue at the area where the RPE was lost^17,25^. The lack of repair was instead considered as zones denuded of RPE or SHT with damaged outer retinal layers directly attached to the Bruch’s membrane (Fig. 1).

Tears were then categorized according to the Sarraf’s grading system^26^. and as early or late tears whether they occurred before or after six months from starting the anti-VEGF treatment^8^. The presence, size and location of sub-macular hemorrhages (SMH) at the time of the tear were also evaluated from multimodal imaging assessment and these cases were defined as hemorrhagic tears^27^.

### Imaging analysis

SD-OCT scans and BAF images were analyzed at the time when the RPE tear was diagnosed—considered baseline—and then 6 months and 12 months later. When available, the 18 months follow-up was also included in the analysis. Visits performed within two months before or after the abovementioned timeframes were considered acceptable, given the retrospective nature of the study.

OCT scans were inspected for the presence of repair tissue by two masked graders unaware of the study aims (FR and SP). All the B-scans including the repair tissue were exported as high-resolution (.tiff) images from the SD-OCT software (Eye Explorer Version 1.10.4.0, Heidelberg Engineering, Heidelberg, Germany) and then imported into ImageJ 1.50 (National Institute of Health; Bethesda, USA). The borders of the whole repair tissue including the RPE and the SHT (or both) were manually outlined by the same masked investigators using the free-hand selection tool.

**Figure 2:**
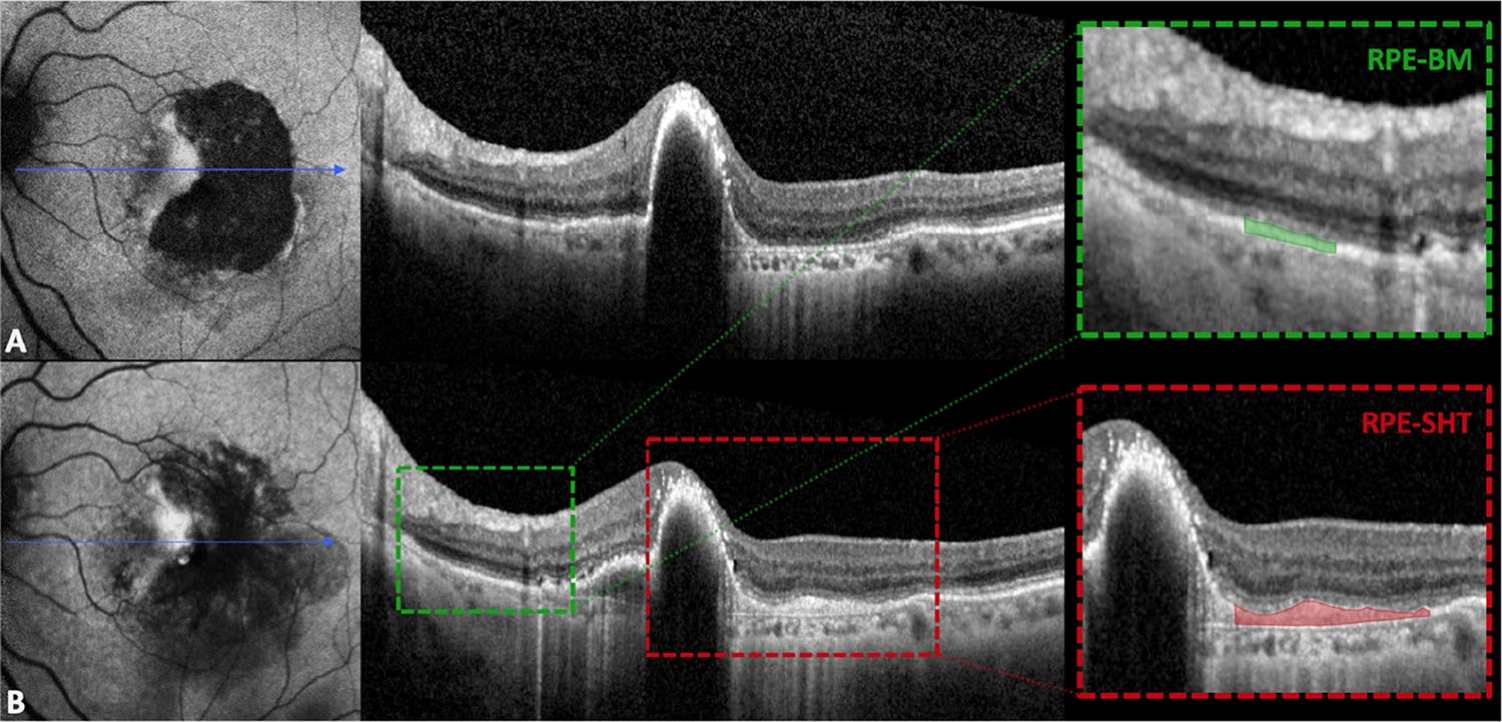
Measurement of repair tissue reflectivity following retinal pigment epithelial (RPE) tear by means of spectral-domain optical coherence tomography (SD-OCT). (**A**) and (**B**) show blue-light autofluorescence and SD-OCT scans of a patient at baseline and at 12 months of follow-up, respectively. Red dashed box highlights the formation of the RPE-subretinal hyperreflective tissue complex that was quantitatively measured after manual delineation. RPE-Bruch’s membrane complex, used as internal reference, is presented in the green dashed box.

The corresponding SD-OCT scan at the time of the RPE tear was taken as reference to identify the borders of the repair tissue more accurately. The reflectivity of the RPE-SHT complex was objectively quantified measuring the mean gray value of the selected pixels area (ranging from 0 to 255). Similarly, the reflectivity of a 500-μm area of RPE-Bruch’s membrane in the proximity of the RPE tear but unaffected by the MNV lesion or by possible masking effects (e.g., retinal vessels or exudates) was used as internal reference for each B-scan. The relative reflectivity (RR) of the repair tissue was obtained by averaging the ratio between the mean gray values of the RPE-SHT complex and those of the internal sample (e.g., RPE-Bruch’s membrane distant from MNV) collected for all B-scans encompassing the affected area, as already described in other studies^28,29^. Differently from other reflectivity studies, the RPE-Bruch’s membrane complex was preferred as reference to the external limiting membrane and the inner nuclear layer as these layers might be altered in nAMD by sub-retinal and intra-retinal exudation^30^. Figure 2 thoroughly illustrates the methodology adopted to quantify the RR.

Blue-light fundus autofluorescence images were qualitatively assessed for recovery of BAF signal in the area where the tear occurred by two masked graders (AI and MBP). BAF signal recovery was defined as shrinking of the hypo-autofluorescent area or as an increase in the brightness of the involved area.

### Statistical analysis

Descriptive statistics included the mean, standard deviation, median, quartiles (Q1 and Q3) and percentages where appropriate.

The following variables were included in our analysis: age at the time of RPE tear appearance, sex, type of MNV, grade and type (early or late) of RPE tear, presence of SMH at the time of tear, extent of follow-up, RR of the RPE-SHT complex, recovery of BAF signal, number of intravitreal injections received, BCVA at baseline, 6 months, 12 months and at 18 months of follow-up (if available).

The differences between baseline and follow-up values were explored with Student’s T test, Pearson’s correlation test and Fisher’s exact test. Inter-observer reproducibility for the four masked investigators (FR and SP; AI and MBP) was tested using intra-class correlation coefficients and k-factors (ICC and k, respectively; 95% confidence intervals) for quantitative and qualitative variables, respectively.

The effect of all the collected variables were tested using univariate linear regression to identify the ones that significantly affected the final visual acuity. Those that resulted significant in the univariate analysis were then analyzed using a multivariate analysis. The same approach was used to investigate the effect of all included variables on the RR of the RPE-SHT complex.

All statistical analyses were performed using R language (version 3.4.1, R Development Core Team). Statistical significance was set at *p* < 0.05.

## Results

Forty-nine eyes form 46 patients were included in the study. The mean age was 76.1 ± 7.0 years (range: 60–90 years) and 19 patients were males. The mean BCVA at the time of RPE tear was 0.54 ± 0.27 LogMAR (range: 0.20–1.10 LogMAR, approximately 20/70 Snellen equivalent). The majority of our cases were affected by large RPE tears (grades 3–4, 73%). Twenty-one eyes (43%) had signs of SMH at onset. The vast majority (90%) of these showed small-sized (between 1 and 4 disk diameters) sub-macular hemorrhages that were prevalently located in extra-foveal areas (76%).

The mean follow-up period was of 16.3 ± 3.0 months (range: 12–19 months) with 35 eyes (71%) having 18 months of follow-up. Thirty-eight eyes (78%) showed some sort of healing process during the follow-up characterized by the presence of RPE-SHT complex on OCT. These eyes had significantly better BCVA at the end of the follow-up compared to eyes that lacked signs of repair (0.59 ± 0.32 vs. 0.87 ± 0.33 LogMAR; t = 1.98, *p* = 0.041). Likewise, eyes without signs of SMH showed significantly higher final BCVA compared with those with SMH at rip onset (0.42 ± 0.26 vs. 0.95 ± 0.34 LogMAR; t = 3.91, *p* < 0.001).

No significant differences in the distribution of the grade of tears, early or late onset, MNV type, SMH presence at baseline, age, sex and eye laterality were found between eyes with and without tissue repair (all *p* > 0.05). Complete demographic and clinical characteristics of the two groups are reported in Table 1.

**Table 1:** Demographic and clinical characteristics of the studied cohort.

	Total	Repaired	Unrepaired	*p* values
**N (%)**	49	38 (78%)	11 (22%)	
**Age (years)**	76.1 ± 7.0	76.3 ± 7.0	75.9 ± 8.9	0.89
**Sex (M/F)**	19/30	17/21	2/9	0.08
**Eye (R/L)**	23/26	18/20	5/6	0.70
**Follow-up (months)**	16.3 ± 3.0	16.5 ± 3.2	16.0 ± 2.6	0.63
**BCVA (LogMAR)**				
At RPE tear	0.54 ± 0.27	0.52 ± 0.26	0.62 ± 0.33	0.37
At follow-up	0.66 ± 0.37	0.59 ± 0.32	0.87 ± 0.33	0.04
**No. of injections**	7.4 ± 3.4	7.4 ± 3.6	7.2 ± 2.9	0.60
**SMH at baseline (%)**	21 (43%)	16 (42%)	5 (45%)	0.76
**Tear onset**				
Early (%)	33 (67%)	25 (66%)	8 (73%)	0.40
Late (%)	16 (33%)	13 (34%)	3 (27%)	
**Tear grade**				
Grade 1	4 (8%)	4 (11%)	0 (0%)	0.93
Grade 2	9 (18%)	7 (18%)	2 (18%)	
Grade 3	19 (39%)	13 (34%)	6 (55%)	
Grade 4	17 (35%)	14 (37%)	3 (27%)	
**MNV type**				
Type 1 (occult)	37 (76%)	30 (79%)	7 (64%)	0.21
Type 2 (classic)	0 (0%)	0 (0%)	0 (0%)	
Type 3 (RAP)	12 (24%)	8 (21%)	4 (36%)	
**Anti-VEGF used**				
Bevacizumab	15 (31%)	11	4	0.86
Ranibizumab	21 (43%)	17	4	
Aflibercept	13 (26%)	10	3	

Legend: N, number; BCVA, best-corrected visual acuity; SMH, sub-macular hemorrhage; MNV, macular neovascularization; RAP, retinal angiomatous proliferation; VEGF, vascular endothelial growth factor.

### BCVA analysis

Univariate analysis demonstrated that final BCVA was significantly and positively influenced by the number of injections received (*p* = 0.012). The same approach revealed a negative correlation between final BCVA and RR of the repair tissue, age, grade of the tear, length of follow-up and the presence of a hemorrhagic RPE tear (all *p* < 0.05). When assessed with multivariate analysis, final BCVA appeared to be significantly affected only by the reflectivity of the RPE-SHT complex (*p* < 0.01), the number of injections received (*p* < 0.001) and the presence of a hemorrhagic tear at baseline (*p* < 0.001).

### RR analysis of the repair tissue

**Figure 3:**
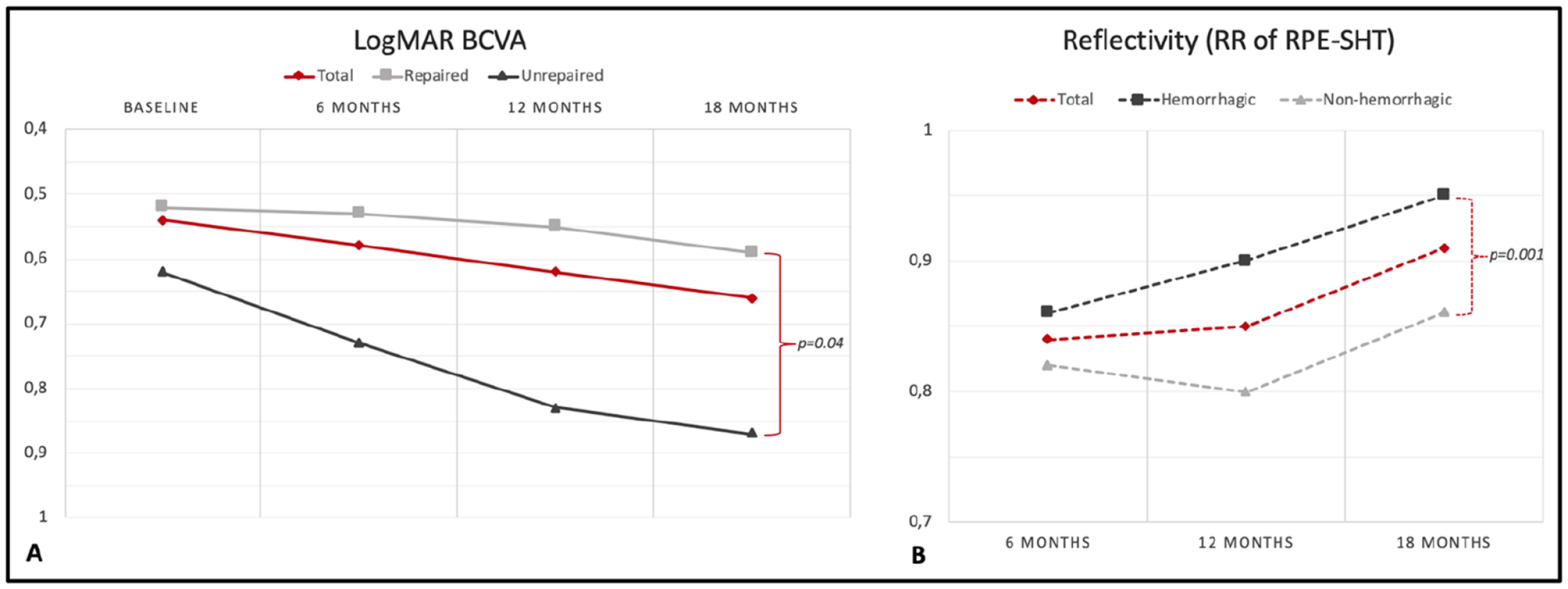
Variation of the best-corrected visual acuity (BCVA) and of the relative reflectivity (RR) of the repair tissue over the follow-up. (**A**) shows the visual acuity changes occurring in time; BCVA appears to deteriorate remarkably in patients that did not have signs of repair tissue formation (*p* = 0.04). The reflectivity of the repair tissue (**B**) increases in time during the follow-up particularly in patients with hemorrhagic tears at baseline (*p* = 0.001).

A higher relative reflectivity of the RPE-SHT complex strongly and negatively correlated with the final BCVA (r =  − 0.68, *p* < 0.001). The RR progressively and significantly increased over time (*p* = 0.002) and a mild, but significant association was observed between the recovery of BAF signal and a lower RPE-SHT complex RR (0.87 ± 0.09 vs. 0.94 ± 0.09; t = 2.1, *p* = 0.04). Inter-observer variability was acceptable for RR of the repair tissue and BAF signal recovery (ICC = 0.921 [0.897–0.943] and k = 0.85 [0.78], *p* < 0.05, respectively). Changes in BCVA and RR of the RPE-SHT complex over time are shown in Fig. 3.

Univariate linear regression analysis revealed that RR of the RPE-SHT complex positively correlated with age of the patients, the hemorrhagic tears and with time (all *p* < 0.05). On multivariate analysis, only hemorrhagic tears (*p* < 0.001) and time (*p* < 0.01) were confirmed to significantly and positively affect the reflectivity of the RPE-SHT complex.

**Figure 4:**
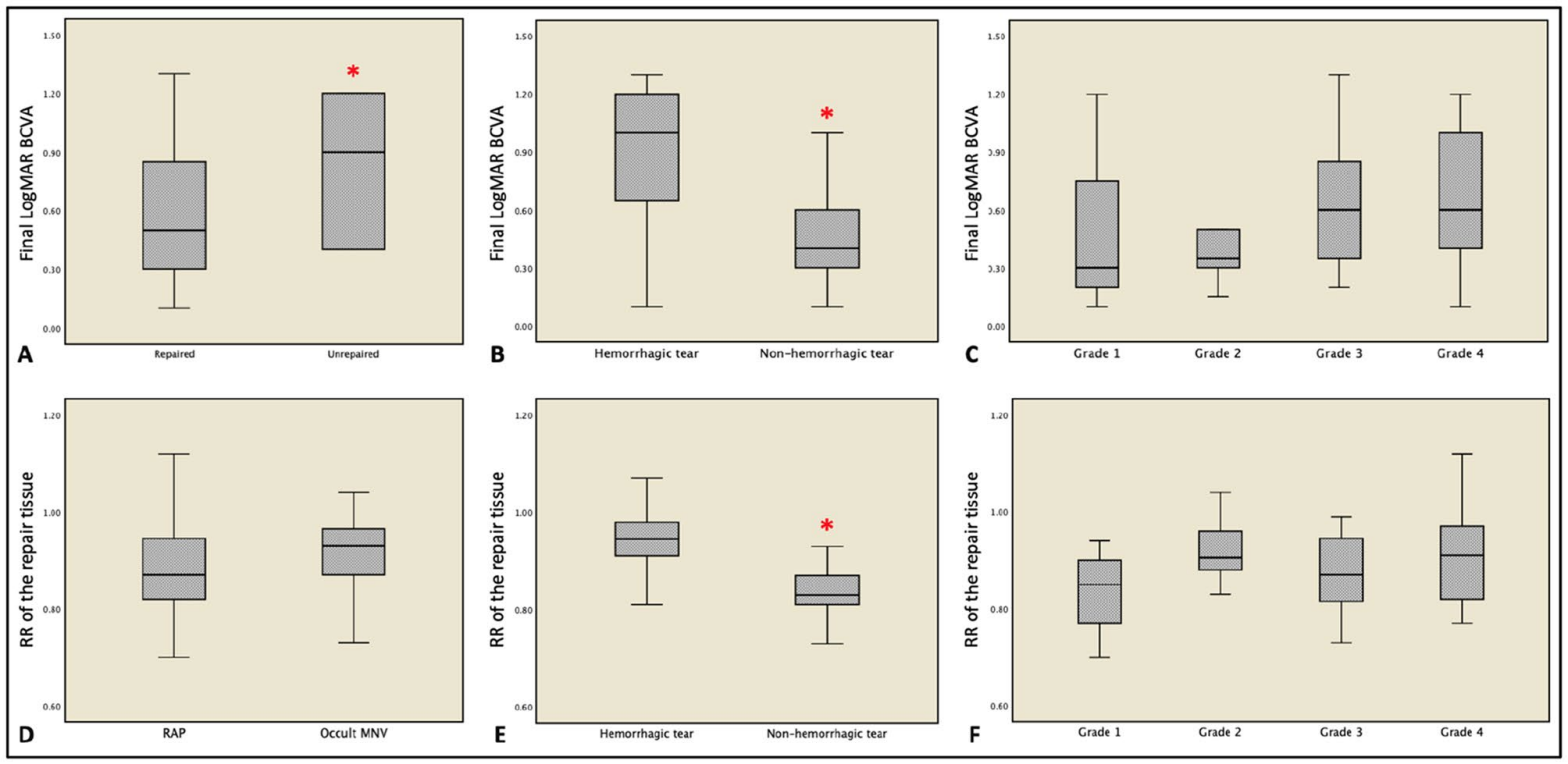
Visual acuity (**A**–**C**) and reflectivity of the repair tissue (**D**–**F**) in different patients’ subgroups. Final visual acuity was significantly lower in patients that did not show signs of healing over the follow-up (**A**) and in those cases characterized by a hemorrhagic rip at baseline (**B**). The identification of sub-retinal hemorrhage was also associated with a higher reflectivity of the repair tissue (**E**). Red asterisks indicate statistical significance (*p* < 0.05).

**Figure 5:**
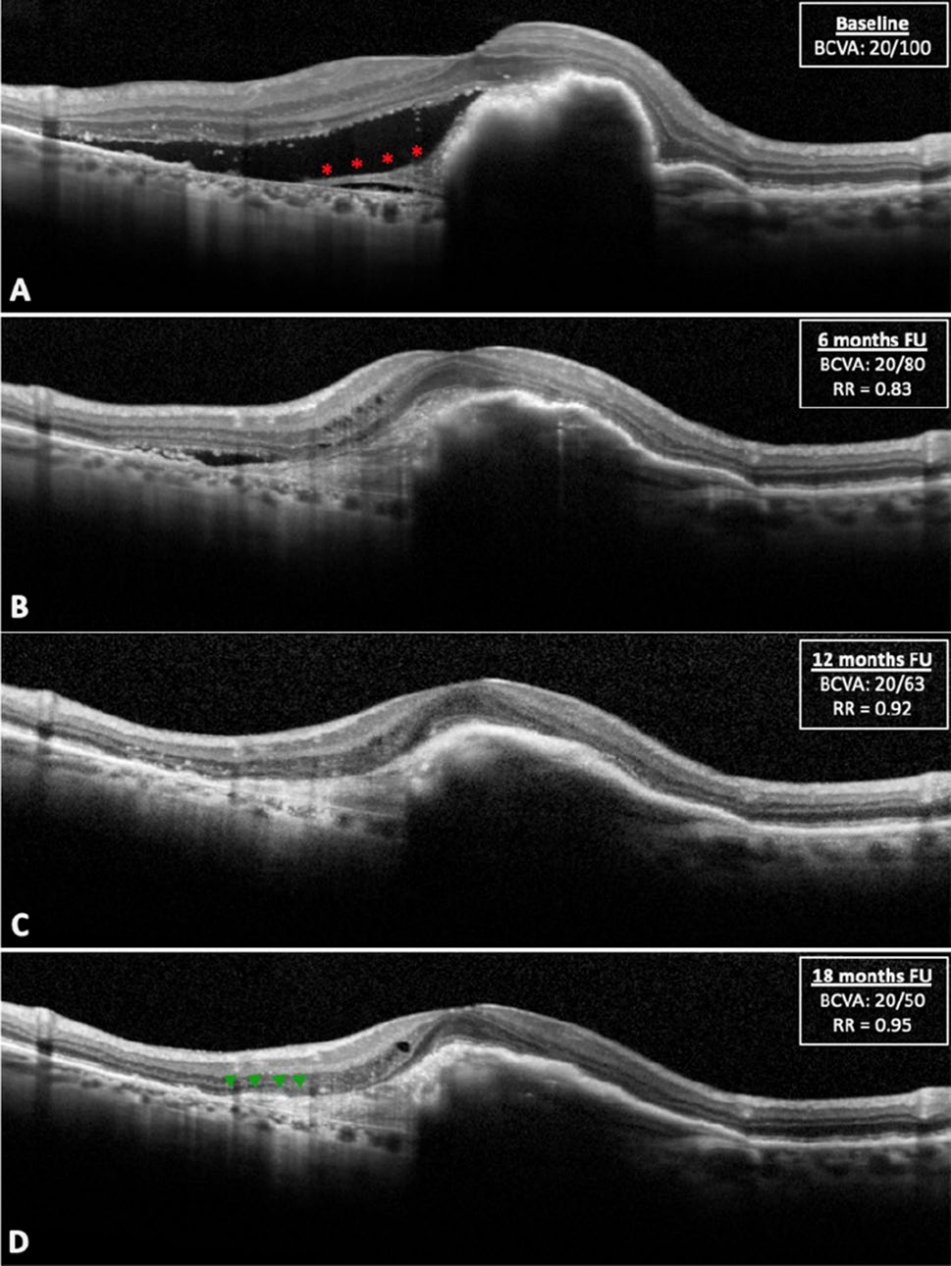
Formation and remodeling of the repair tissue on optical coherence tomography (OCT) over 18 months of follow-up. (**A**) shows a large retinal pigment epithelial (RPE) tear involving the fovea (grade 4) with several folds of the retracted RPE and direct exposure of the Bruch’s membrane; an initial proliferative tissue can be appreciated (red asterisks). After 6 months (**B**), some sort of subretinal hyperreflective tissue (SHT) almost completely covers the bed of exposed Bruch’s membrane. Over the follow-up, the relative reflectivity RR) of the repair tissue gradually increases (**C**) and in occasion of the last visit a distinct RPE layer covering the SHT can be noticed (**D**, green arrowheads).

No associations were found with sex and eye laterality (all *p* > 0.05, range: 0.24–0.48). Figure 4 shows the behavior of final visual acuity and repair tissue reflectivity according to different clinical variables. An exemplifying case of repair tissue formation following RPE tear during the follow-up is reported in Fig. 5.

## Discussion

In this retrospective study, we analyzed the OCT characteristics of the repair tissue following RPE tears secondary to anti-VEGF treatment for nAMD. We found that the great majority of eyes showed some sign of healing over 18 months of follow-up. A higher reflectivity of newly growing RPE layer and subretinal proliferative tissue on OCT correlates with worse visual outcomes.

Significant visual loss is a major concern in case of RPE tear occurrence, especially when the macular area is extensively involved or when the number of anti-VEGF injections administered during the first year of follow-up is low^2–4,8,9,26^. Different repair processes have been described in eyes experiencing a tear including re-attachment of the ruptured RPE, migration of new hypopigmented RPE cells and the formation of a fibrous plaque^9–17^. However, to predict visual outcomes from multimodal imaging assessment remains a challenge in these eyes. Unfortunately, the current OCT technology does not allow to recognize the different components of the healing tissue. For this reason, we decided to study the mean relative reflectivity of the whole repair tissue (RPE-SHT complex) and to assess its possible correlations with visual outcomes and other clinical and imaging findings.

Our results show that visual acuity was significantly higher in eyes that showed some sort of repair process as previously reported by Heimes et al.^9^. Among these, the final BCVA was negatively and independently correlated both with a higher reflectivity of the RPE-SHT complex and with the presence of a sub-macular hemorrhage at the time of the tear. This finding may be explained by the toxic effect exerted by sub-retinal blood on photoreceptors and by the consequent development of a fibrotic plaque^23,24^. On the other hand, vision seems to be in part preserved by a higher frequency of intravitreal injections, confirming the data already described in literature^3,7,8,31^.

The reflectivity of the repair tissue was found, instead, to be significantly affected by the occurrence of a hemorrhagic rip and by time. These relationships suggest that sub-macular hemorrhage particularly favors the development of a fibrotic component in the repair tissue, while a progressive remodeling process ensues over time^32,33^. The higher reflectivity of the RPE-SHT complex at the last follow-up visit may in fact reflect the natural progression of the fibrotic changes as well as the gradual pigmentation of the newly-growing RPE layer^25,34^. On the other hand, a lower reflectivity of the repair tissue was significantly associated with the recovery of autofluorescence signal over time, seeming to suggest the presence of a hypopigmented RPE layer filling the previous tear defect^12,16^.

According to our data final visual acuity and reflectivity of the RPE-SHT complex were not affected by other clinical characteristics, including the type of MNV, the grade and the onset (early or late) of RPE tear. This might be explained by the fact that visual outcomes also depend on the progressive nature of macular degeneration and that some patients, despite the foveal involvement, are able to develop new loci of fixation during the months following the RPE tear^8,35^.

We acknowledge that our study has several limitations, especially considering the small sample size. The delineation of the repair tissue on OCT and the assessment of BAF images were performed manually thus they both may be affected by human error even if inter-observer variability demonstrated concordant measurements. Other potential limitations included the relatively limited follow-up, the retrospective design of the study and the lack of data from functional tests (e.g., microperimetry or multifocal electroretinogram) other than BCVA that would provide a better characterization of the retinal function.

Moreover, we did not directly measure the distance of the tear from the fovea as this can potentially affect the BCVA. However, we classified the lesions according to Sarraf’s grading system that is based on rip size and fovea involvement and is known to correlate with visual function^26^. Lastly, we preferred to analyze logarithmic-displayed OCT scans rather than raw images to measure the RR of the repair tissue. This approach could be less accurate as it prevented us from assessing the actual non-transformed signal data but it made the results of our study easily reproducible from any clinician^36^.

In essence, our study explored the relationship between visual outcomes and the reflectivity of the healing tissue developing after RPE tears in patients with nAMD. At 18 months of follow-up, visual acuity seems to be negatively affected by a higher reflectivity of the RPE-SHT complex and by the presence of a hemorrhagic tear. These results could provide clinicians with adjunctive information other than the tear extension to predict functional outcomes in eyes experiencing an RPE tear.
